# Optimizing lipocalin sequence classification with ensemble deep learning models

**DOI:** 10.1371/journal.pone.0319329

**Published:** 2025-04-16

**Authors:** Yonglin Zhang, Lezheng Yu, Li Xue, Fengjuan Liu, Runyu Jing, Jiesi Luo

**Affiliations:** 1 Department of Pharmacy, Affiliated Hospital of North Sichuan Medical College, Nanchong, Sichuan, China; 2 School of Chemistry and Materials Science, Guizhou Education University, Guiyang, China; 3 School of Public Health, Southwest Medical University, Luzhou, China; 4 School of Geography and Resources, Guizhou Education University, Guiyang, China; 5 School of mathematics and big data, Guizhou Education University, Guiyang, China; 6 School of Basic Medical Sciences, Southwest Medical University, Luzhou, Sichuan, China; 7 Sichuan Key Medical Laboratory of New Drug Discovery and Druggability Evaluation, Luzhou Key Laboratory of Activity Screening and Druggability Evaluation for Chinese Materia Medica, Southwest Medical University, Luzhou, Sichuan, China; Emory University, UNITED STATES OF AMERICA

## Abstract

Deep learning (DL) has become a powerful tool for the recognition and classification of biological sequences. However, conventional single-architecture models often struggle with suboptimal predictive performance and high computational costs. To address these challenges, we present EnsembleDL-Lipo, an innovative ensemble deep learning framework that combines Convolutional Neural Networks (CNNs) and Deep Neural Networks (DNNs) to enhance the identification of lipocalin sequences. Lipocalins are multifunctional extracellular proteins involved in various diseases and stress responses, and their low sequence similarity and occurrence in the ‘twilight zone’ of sequence alignment present significant hurdles for accurate classification. These challenges necessitate efficient computational methods to complement traditional, labor-intensive experimental approaches. EnsembleDL-Lipo overcomes these issues by leveraging a set of PSSM-based features to train a large ensemble of deep learning models. The framework integrates multiple feature representations derived from position-specific scoring matrices (PSSMs), optimizing classification performance across diverse sequence patterns. The model achieved superior results on the training dataset, with an accuracy (ACC) of 97.65%, recall of 97.10%, Matthews correlation coefficient (MCC) of 0.95, and area under the curve (AUC) of 0.99. Validation on an independent test set further confirmed the robustness of the model, yielding an ACC of 95.79%, recall of 90.48%, MCC of 0.92, and AUC of 0.97. These results demonstrate that EnsembleDL-Lipo is a highly effective and computationally efficient tool for lipocalin sequence identification, significantly outperforming existing methods and offering strong potential for applications in biomarker discovery.

## Introduction

Lipocalins are a subgroup within the larger calycin family, a class of small secreted proteins known for their affinity for hydrophobic molecules. They are found across all kingdoms of life except Archaea [1]. These proteins typically range in length from 165 to 200 amino acid residues and have a molecular weight of approximately 20 kDa [2, 3]. Despite generally exhibiting low amino acid sequence identity (usually not exceeding 30%), lipocalins are characterized by three structurally conserved regions (SCRs) and share significant similarities in their three-dimensional structures [4,5]. Their sequences and distribution are highly diverse, and they play pivotal roles in a variety of biological processes, including stress and immune responses, retinoid binding, pheromone transport, prostaglandin synthesis, tumorigenesis, and apoptosis [6–12]. Certain lipocalins, such as ɑ1-microglobulin [13], apolipoprotein D, complement C8 gamma, lipocalin 2 [14], orosomucoid, protein HC, prostaglandin D synthase, retinol-binding protein, and tear lipocalin [15]—have been identified as biomarkers for various diseases. Consequently, there is an urgent need for efficient and accurate methods to identify lipocalins, which will aid in understanding their diverse functions and facilitate the development of novel therapeutics.

Accurately identifying and classifying lipocalin proteins is a challenging task in computational bioinformatics due to their structural and functional diversity, as well as the complex relationships between their sequences. Traditional experimental approaches for protein classification are labor-intensive and cannot keep pace with the growing volume of sequence data generated by high-throughput sequencing technologies. Computational methods, particularly those based on machine learning, offer a scalable solution to this challenge. However, the development of reliable computational models for lipocalin classification is hindered by the limited availability of annotated datasets, potential biases in feature selection, and the need for robust algorithms capable of generalizing across diverse lipocalin families. Muthukumar and colleagues utilized MALDI-TOF/MS to validate the presence of a 14.5 kDa lipocalin protein in the urine of female rodents, establishing a correlation between its expression in urine and the phases of the estrous cycle [16]. Yao *et al*. conducted a detailed characterization of rLcn13, a member of the rat epididymal lipocalin family [17]. The identification of the rLcn13 lipocalin protein involved various experimental procedures, including breeding white mice, cloning serum, immunohistochemical staining, and reverse transcription quantitative PCR (RT-qPCR). To reduce time and infrastructure costs, several computational methods employing machine learning algorithms have been developed as more accessible solutions for lipocalin identification. Ramana and Gupta employed a support vector machine (SVM) approach named LipocalinPred, utilizing amino acid composition (AAC), dipeptide composition (DPC), secondary structure composition (SSC), and position-specific scoring matrix (PSSM) as input features [18]. The integrated features of PSSM and SSC produced the best model, with an overall accuracy of 90.72%, sensitivity of 88.97%, and specificity of 92.16%. Pugalenthi *et al*. introduced an SVM-based tool, LipoPred, demonstrating its effectiveness in predicting lipocalin proteins, achieving an accuracy of 88.61%, sensitivity of 89.26%, specificity of 85.27%, and Matthews correlation coefficient (MCC) of 0.74 [19]. Nath and Subbiah leveraged diverse balanced training sets and classifier fusion schemes to enhance prediction performance [20]. Using Random Forest (RF) and K-nearest neighbor (KNN) classifiers, along with AAC, attribute group composition, and rationalized n-grams as features, they achieved high performance on test sets. Zulfiqar *et al*. utilized an RF-based approach, incorporating six types of features to predict lipocalins, achieving an impressive accuracy of 95.03% and an area under the curve (AUC) of 0.987 during 10-fold cross-validation [21]. While the traditional machine learning algorithms mentioned have shown promise, exploring improved alternative models remains essential for further enhancing predictive performance.

Recently, deep learning (DL), a significant sub-discipline of machine learning, has been successfully applied to the identification and classification of various biological sequences [22, 23]. For example, StackedEnC-AOP, which employs stacked ensemble techniques, achieves high predictive performance for antioxidant peptides, demonstrating the potential of combining multiple architectures [24]. A novel approach, DeepAVPTPPred, applies deep learning to predict antiviral peptides (AVPs) using sequence-based features, achieving high accuracy and generalization [25]. Additionally, an innovative stacked ensemble deep learning approach has been tailored for predicting antiviral peptides, further demonstrating the utility of stacked models in complex biological sequence classification tasks [26]. Applications of deep learning in genomics include transcription factor binding, DNase sensitivity, CpG methylation, and predicting the effects of genetic variation on gene regulatory mechanisms, such as DNA accessibility and splicing [27]. In proteomics, deep learning plays a crucial role in predicting protein structure, classifying protein sequences, determining protein subcellular localization, and identifying peptides. Therefore, constructing DL models to enhance the predictive performance of lipocalin protein classification is of substantial interest. DL model architectures primarily include convolutional neural networks (CNNs), recurrent neural networks (RNNs) with bidirectional long short-term memory (BiLSTM) or bidirectional gated recurrent units (BiGRU), and combinations of these networks (e.g., CNN-BiLSTM and CNN-BiGRU). Classical deep neural networks (DNNs), evolved from artificial neural networks (ANNs), typically utilize sequence, structure, function, and other features as input. Several studies have reported that ensemble frameworks incorporating different DL architectures tend to achieve superior predictive performance compared to single architectures [28–30].

In this study, we address these challenges by developing a deep learning-based framework with tailored feature selection and sequence encoding strategies to enable accurate and interpretable classification of lipocalin sequences. We employed Convolutional Neural Network (CNN) and Deep Neural Network (DNN) architectures to construct the ensemble framework, EnsembleDL-Lipo, for the precise identification of lipocalins from their primary sequences. The CNN architecture utilized a dictionary encoding method to extract protein sequence information, while the DNN architecture employed nine PSSM-based features to represent protein sequences. A total of 511 unique deep learning models were generated through permutations, and their performance in lipocalin recognition was evaluated, with particular emphasis on the top ten models exhibiting exceptional results. By integrating these individual models with varying input features, we developed the ensemble deep learning model, combining a CNN model with dictionary encoding and several DNN models using three specific PSSM-based features (DFMCA_PSSM, DPC-PSSM, and PSSM-AC). To ascertain the most effective approach for lipocalin recognition, the performance of a single high-accuracy deep learning model was compared with the ensemble deep learning framework. The efficiency of the ensemble method in identifying lipocalin proteins was assessed using a training dataset consisting of 212 positive samples and 211 negative samples, alongside an independent test dataset containing 42 lipoproteins and 53 non-lipoproteins. Finally, to demonstrate the superior discriminatory capabilities of the ensemble approach, its performance metrics, such as accuracy (ACC), *F*-value, recall, precision (PRE), and Matthews correlation coefficient (MCC), were compared with those of the previously established Lipo-RF and LipocalinPred models. We propose a tailored feature selection strategy, systematically evaluating 511 feature combinations to identify the optimal biologically relevant feature set, and integrate an ensemble learning framework to enhance robustness and generalization across diverse lipocalin sequences. Our model is benchmarked against existing approaches, demonstrating its state-of-the-art performance and practical utility in bioinformatics applications.

## Materials and methods

### Generation of training and test dataset

Accurate and reliable deep learning prediction models rely on high-quality datasets. The dataset curated by Zulfiqar and colleagues (available at https://zenodo.org/record/5844993#.YeAL7fgRVPZ) serves as an example of a large, balanced dataset meticulously selected for training and testing deep learning models designed to identify lipocalins [21]. A total of 614 protein sequences, equally divided between 307 lipocalins and 307 non-lipocalins, were sourced from the UniProt database. An identity threshold of 40% was set, and redundant sequences were removed using the CD-HIT method [31]. The resulting training dataset consisted of 212 positive and 211 negative samples. The performance of the developed deep learning models was evaluated using independent test datasets, which included 42 lipoproteins and 53 non-lipoproteins.

### Feature encoding schemes

This study aims to develop an ensemble deep learning approach for the accurate and efficient identification of lipocalin proteins from sequence data. A standardized dataset is essential for the effective extraction of relevant features from these sequences. To identify the optimal feature set for encoding lipocalin protein sequences, we employed a dictionary encoding method in combination with nine PSSM-based features.

### Dictionary encoding

Each protein sequence is encoded numerically, with the 20 natural amino acids represented by integers from 1 to 20 (e.g., alanine as 1) [32], while unknown or pseudo-amino acids are encoded as 0. This method transforms each protein sequence into an *L*-dimensional vector, where *L* is the sequence length.

### Evolutionary information-based features

Previous research has consistently demonstrated that incorporating evolutionary information significantly enhances the performance of classifiers in protein recognition [33–35]. To enhance the classification performance for protein recognition, we incorporated evolutionary information by leveraging the position-specific scoring matrix (PSSM) [36], which captures evolutionary patterns within protein sequences. Using the PSI–BLAST program [37], homologous sequences were searched in the Swiss-Prot or NCBI non-redundant protein databases, followed by multiple sequence alignment to generate an initial PSSM matrix. The matrix rows correspond to amino acid residue positions, columns denote residue names, and values represent the binary logarithms of residue frequencies from the alignments. Positive values indicate conserved residues, while negative values indicate non-conserved ones. Various matrix transformations were applied to extract nine PSSM-based features. These features were calculated using the PSSMCOOL package, with details available at PSSMCOOL documentation [38].

### Single deep learning model architectures

The study explored the feasibility of two distinct deep learning architectures by employing 10 individual models to differentiate lipocalin proteins. In the CNN architecture, protein sequences were encoded using an amino acid dictionary representation, and a probability score between 0 and 1 was computed to classify lipocalins. In the DNN architecture, nine PSSM-based features were used as input, resulting in the development of nine distinct DNN models.

### Ensemble deep learning framework design

In recent years, ensemble deep learning frameworks have gained significant popularity for analyzing various biological sequences due to their superior predictive capabilities compared to individual model architectures [39–41]. The ensemble deep learning model developed in this study integrates two primary architectures with diverse input features. Protein sequences of lipocalins are encoded using an amino acid dictionary embedding representation, which serves as the foundation of the model. This encoded representation is then processed by convolutional layers to capture both local sequence information and global sequence patterns. Discrete feature sets, such as PSSM-based descriptors, are processed by the DNN layer, where all feature sets are aligned to identical dimensions. Through a flattening operation, sequential features are transformed into one-dimensional arrays for each sample. The core architecture of the ensemble deep learning framework includes CNN and DNN, with various combinations of PSSM features enhancing the model’s performance up to the final fully connected layer. The 511 DNN models integrated into the core architecture utilize a combination of PSSM features, including AAC-PSSM, DP-PSSM, DPC-PSSM, Pse-PSSM, PSSM-AC, PSSM400, SVD_PSSM, Single_Average, and DFMCA_PSSM. To improve the reliability of the model, each training iteration is repeated five times. This approach reduces potential errors caused by random factors, thereby minimizing sampling and model fitting inconsistencies.

### Implementation

All single deep learning models and our ensemble deep learning framework were designed, trained, and assessed using the autoBioSeqpy tool [42]. The commands were executed on a Windows 10 workstation equipped with an NVIDIA GeForce RTX GPU and CUDA 10.2.95.

### Performance assessment

To comprehensively assess the performance of our ensemble deep learning framework, five metrics commonly used in the field of bioinformatics and computational biology are adopted in this study. They are accuracy (ACC), *F*-value, Recall, precision (PRE), and Matthew’s correlation coefficient (MCC), and defined as follows:


$$ACC=\frac{TP+TN}{TP+FP+TN+FN}$$ (1)

$$F-value=2\times \frac{TP}{2TP+FP+FN}$$ (2)

$$Recall=\frac{TP}{TP+FN}$$ (3)

$$PRE=\frac{TP}{TP+FP}$$ (4)

$$MCC=\frac{\left(TP\times TN\right)-\left(FN\times FP\right)}{\sqrt{\left(TP+FN\right)\times \left(TN+FP\right)\times \left(TP+FP\right)\times \left(TN+FN\right)}}$$ (5)

where *TP*, *TN*, *FP*, and *FN* represent the numbers of true positive, true negative, false positive, and false negative, respectively.

In addition, the receiver operating characteristic (ROC) curves are selected for a visible performance comparison. As two key quantitative indexes of the overall performance, the areas under the ROC curve (AUC) and the precision-recall (PR) curve are also computed, and shown by the ROC and PR plots respectively.

## Results and discussion

### Performance comparison of different single deep learning models

**Fig 1:**
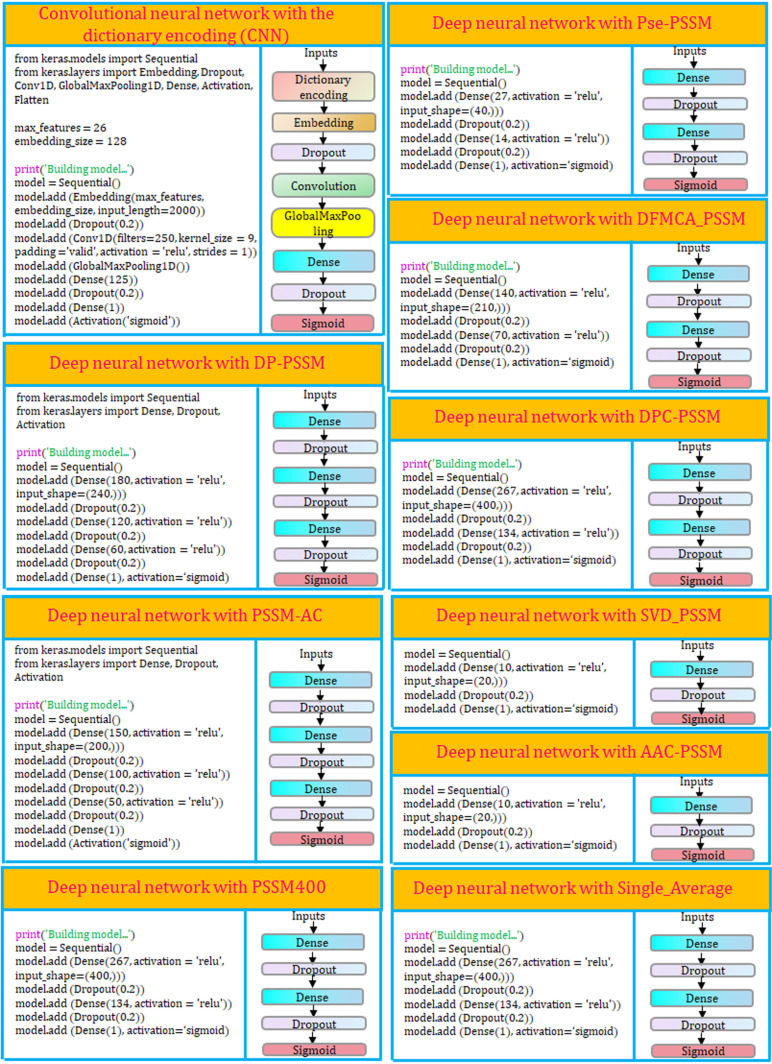
Overview of all deep learning (DL) architectures, comprising one Convolutional Neural Network (CNN) and nine Deep Neural Network (DNN) model configurations.

**Fig 2:**
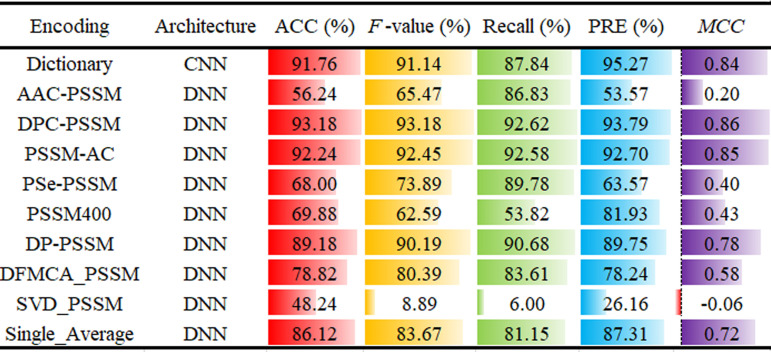
Performance comparison of different deep learning models on the training dataset.

The ten distinct single deep learning models were analyzed, comprising one CNN model and nine DNN models (AAC-PSSM, DFMCA-PSSM, DP-PSSM, DPC-PSSM, Pse-PSSM, AC-PSSM, PSSM400, SVD-PSSM, Single-Average), with the detailed structure of each model depicted in Fig 1. To facilitate robust and equitable comparisons, each model underwent rigorous optimization via hyperparameter tuning (S1 Fig). The comprehensive prediction outcomes of the ten deep learning models on the training dataset are presented in Fig 2. Notably, among the ten deep learning models evaluated, the DNN model with the input feature DPC-PSSM (DNN_DPC-PSSM) exhibited superior predictive performance, achieving an accuracy (ACC) of 93.18%, an *F*-value of 93.18%, a Recall rate of 92.62%, and a Matthews correlation coefficient (MCC) of 0.86. In comparison, the DNN model incorporating PSSM-AC showed slightly lower prediction accuracy, with ACC, *F*-value, Recall, and MCC values of 92.24%, 92.45%, 92.58%, and 0.85, respectively. Following these models, the CNN architecture leveraging dictionary encoding demonstrated relatively strong predictive performance, achieving the highest average precision (PRE) score of 95.27% and ranking third in average accuracy (ACC) (91.76%), *F*-value (91.14%), and MCC (0.84). However, the DNN model with SVD-PSSM as the input feature yielded the poorest average prediction performance, with an average precision of 26.16% and ACC, *F*-value, Recall, and MCC values of 48.24%, 8.89%, 6.00%, and -0.06, respectively. The superior performance of the DNN architecture with DPC-PSSM features can be attributed to the more detailed and informative nature of these features, which provide a richer representation of sequence characteristics. This enables the model to capture more relevant patterns in the lipocalin sequences. In contrast, SVD-PSSM shows poorer performance, likely due to its more compressed feature representation, which may result in a loss of important sequence information, leading to suboptimal predictions.

### Creation and determination of the ensemble deep learning framework

**Fig 3:**
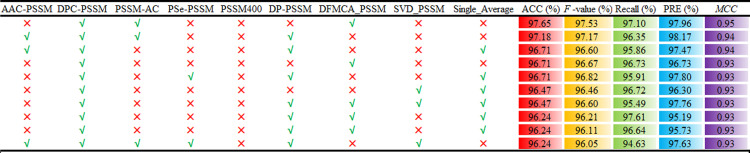
The performance of the top 10 ensemble models on the training dataset.

The ensemble deep learning framework exhibits remarkable superiority in predicting lipocalin proteins, owing to its integration of distinct advantages from diverse deep learning algorithms. Leveraging the CNN core structure and a tandem DNN architecture, we exhaustively explored all feasible combinations of nine PSSM-based features (AAC-PSSM, DFMCA_PSSM, DP_PSSM, DPC_PSSM, Pse_PSSM, PSSM400, PSSM-AC, Single_Average, and SVD_PSSM), culminating in the development of an ensemble comprising 511 deep learning models. The comprehensive predictive results of these ensemble deep learning models on the training dataset are detailed in the S1 Table, with Fig 3 summarizing the prediction performance of the top 10 ensemble models. These outcomes underscore the enhanced performance potential of ensemble deep learning frameworks over single deep learning models, showcasing the varied predictive performance achieved through the fusion of diverse feature inputs.

**Fig 4:**
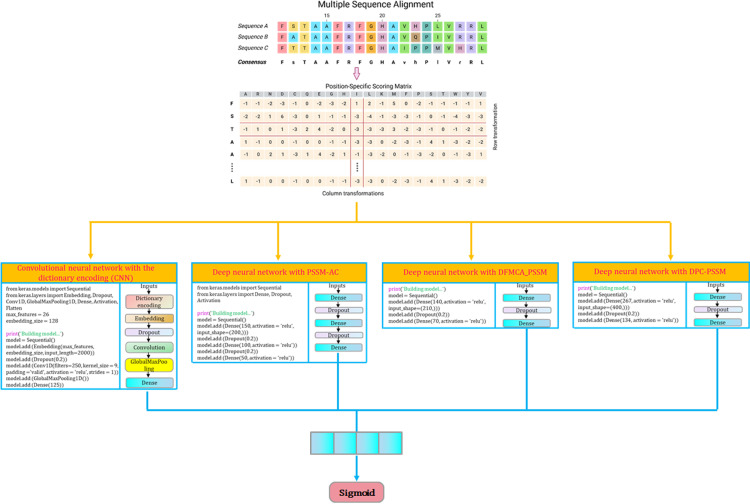
The overview of EnsembleDL-Lipo.

A detailed analysis from the S1 Table reveals that the amalgamated deep learning framework utilizing DFMCA_PSSM, DPC_PSSM, PSSM-AC as input features demonstrated the optimal prediction performance, achieving ACC, *F*-value, recall, precision (PRE), and MCC values of 97.65%, 97.53%, 97.10%, 97.96%, and 0.95, respectively. In contrast, the ensemble framework encompassing all nine features attained an average ACC of 78.84% and an MCC of 0.86, notably lower than the former framework. Among the top ten ensemble models highlighted in Fig 3, five models were constructed based on a combination of four features, two models derived from a combination of three features, and three other models stemmed from combinations of two, five, and six features, respectively. Notably, no deep algorithmic prediction model incorporated more than seven features. It is important to emphasize that an increasing number of PSSM-based feature combinations within the ensemble framework does not always result in superior predictive performance, indicating the presence of redundant information across these features. Ultimately, the deep learning composite framework leveraging the feature combination of DFMCA_PSSM, DPC_PSSM, and PSSM-AC was designated as the ultimate ensemble model for lipocalin protein identification, referred to as EnsembleDL-Lipo (Fig 4).

### Performance evaluation of EnsembleDL-Lipo using the independent test dataset

**Fig 5:**
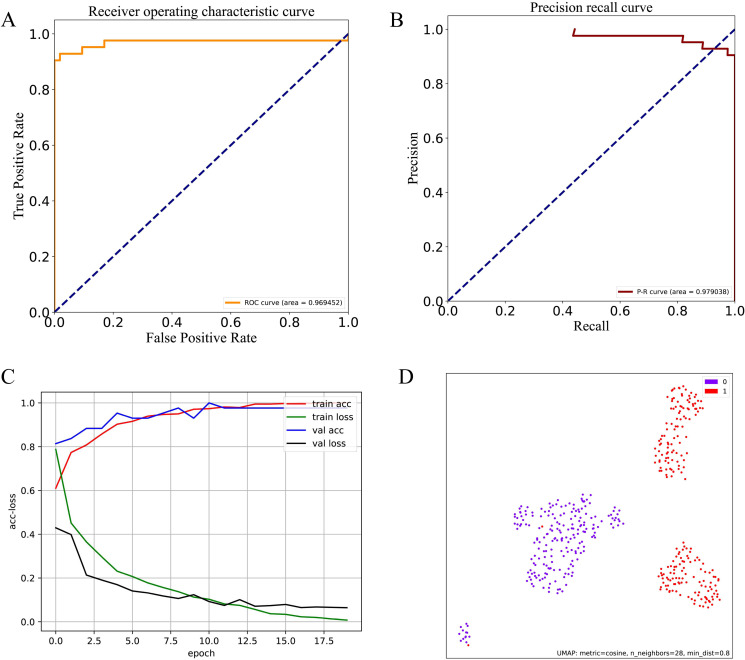
The ROC, PR, accuracy-loss and UMAP of EnsembleDL-Lipo on an independent test set.

The generalization performance of the EnsembleDL-Lipo deep learning framework for predicting lipocalins was further assessed using an independent test dataset comprising 42 lipocalin and 53 non-lipocalin proteins. The results consistently demonstrated the superior predictive capability of the ensemble framework, with only four lipocalins erroneously identified as non-lipocalins, yielding an overall accuracy (ACC) of 95.79%, an *F*-value of 95.00%, a recall of 90.48%, a precision (PRE) of 100.00%, and a Matthews correlation coefficient (MCC) of 0.92. Additionally, the receiver operating characteristic (ROC) and precision-recall (PR) curves were employed to assess the predictive performance of EnsembleDL-Lipo (Figs 5A and 5B). The areas under the ROC and PR curves were calculated as 0.97 and 0.98, respectively. An acc-loss curve (Fig 5C) was generated for further analysis. Furthermore, the layer UMAP technique was utilized to dissect the ensemble deep learning framework (Fig 5D) [43]. The 2D UMAP maps highlight the distinct distribution of lipocalin proteins (red point cloud, label 1) and non-lipocalin proteins (purple point cloud, label 0), with the latter positioned in the latent space of the final hidden layer. To enhance the dispersal of data points in the projection, specific UMAP parameters such as metric, n_neighbors, and min_dist were adjusted to cosine, 28, and 0.8, respectively. It is crucial to recognize that the accurate identification of lipocalin proteins was achieved through the combined features extracted by the ensemble deep learning framework. This empirical evidence underscores the exceptional efficacy and robustness of our proposed ensemble-based deep learning methodology.

### Performance comparison of EnsembleDL-Lipo with the latest methods

In this study, we conducted a comparative analysis of the prediction performance of the EnsembleDL-Lipo deep learning algorithm proposed in this work with the Lipo-RF and Lipocalin-Pred methods for lipocalin proteins. The experimental results of the three methods are summarized in Table 1, utilizing four evaluation metrics: ACC, Recall, MCC, and AUC. The results of the Lipo-RF and Lipocalin-Pred methods were directly obtained from the work of Zulfiqar *et al*. [21]. For the training dataset, the Lipo-RF method achieved ACC, Recall, MCC, and AUC values of 95.03%, 96.20%, 0.90, and 0.99, respectively. Conversely, the EnsembleDL-Lipo approach proposed in this work exhibited superior performance with ACC, Recall, MCC, and AUC values of 97.65%, 97.10%, 0.95, and 0.99, respectively. The enhanced performance of the EnsembleDL-Lipo method is further highlighted on the independent test dataset, although the 91.73% recall obtained by Lipo-RF slightly outperforms the 90.48% achieved by EnsembleDL-Lipo. From the data presented in Table 1, it is evident that the EnsembleDL-Lipo approach significantly improved ACC, MCC, and AUC by 5.89-10.06%, 4.92-14.12%, and 1.35-4.75% compared to Lipo-RF and Lipocalin-Pred, respectively. The effectiveness and utility of the EnsembleDL-Lipo technique for predicting lipocalin proteins were reaffirmed through the comparison of our self-designed protocol with other state-of-the-art methods based on various assessment metrics. These results emphasize the superior predictive capabilities and performance of the EnsembleDL-Lipo deep learning framework in the domain of lipocalin protein identification.

**Table 1:** Performance comparison of EnsembleDL-ATG and two exiting methods on the training and independent test datasets.

	Training dataset				Independent test dataset			
Metrics	ACC	Recall	MCC	AUC	ACC	Recall	MCC	AUC
Lipocalin-Pred	–	–	–	–	85.73	88.41	0.78	0.92
Lipo-RF	95.03	96.20	0.90	0.99	89.90	91.73	0.87	0.96
EnsembleDL-Lipo	97.65	97.10	0.95	0.99	95.79	90.48	0.92	0.97

## Conclusion

In this study, a novel deep learning ensemble model named EnsembleDL-Lipo was developed for the accurate identification of lipocalin proteins. Lipocalins are a diverse group of secreted proteins known for their role in binding and transporting various small hydrophobic molecules, serving as biomarkers for a range of diseases [44–46]. While traditional machine learning algorithms like SVM, RF, and KNN have demonstrated some success in identifying lipocalins, there remains a need to improve predictive performance. The EnsembleDL-Lipo deep learning architecture combines CNN and DNN models to build classifiers, testing 511 different feature combinations. The model utilizing the ‘DFMCA_PSSM+DPC-PSSM+PSSM-AC’ feature set demonstrated the best overall performance on the training dataset, achieving ACC, *F*-value, recall, and MCC scores of 97.65%, 97.53%, 97.10%, and 0.95, respectively. The predictive power of EnsembleDL-Lipo was further validated on an independent test dataset, where it achieved an ACC of 95.79%, an *F*-value of 95.00%, a recall of 90.48%, a precision of 100.00%, and an MCC of 0.92, with only four misclassifications of lipocalins as non-lipocalins. Evaluation through ROC and PR curves yielded impressive area scores of 0.97 and 0.98, respectively, showcasing the robust predictive performance of EnsembleDL-Lipo.

A comparative analysis with existing methods, Lipo-RF and Lipocalin-Pred, revealed the superiority of EnsembleDL-Lipo across both training and independent test datasets, exhibiting improvements in ACC, MCC, and AUC ranging from 5.89% to 10.06%, 4.92% to 14.12%, and 1.35% to 4.75%, respectively. In summary, the proposed EnsembleDL-Lipo deep learning approach offers an efficient computational method for identifying lipocalin proteins, potentially aiding in elucidating their diverse functions and facilitating the discovery of novel therapeutics. The source code and benchmark data for this study are freely available at https://github.com/jingry/autoBioSeqpy/tree/2.0/examples/Lipo.

## Supporting information

S1 Fig Hyperparameter optimization for 10 single-feature deep learning models. (**A**) Performance evaluation of CNN architectures across varying convolutional kernel sizes and filter counts. (**B**) Comparative analysis of DNN architectures using different protein descriptors and hidden layer configurations.
(TIF)

S1 Table Experimental results of the 511 combinations on the training dataset. (XLSX)
