# Supplementary figures and images for: Optimizing lipocalin sequence classification with ensemble deep learning models
Source: PLoS One. 2025 Apr 16;20(4):e0319329. doi: 10.1371/journal.pone.0319329 (PMC12002463; doi:10.1371/journal.pone.0319329)

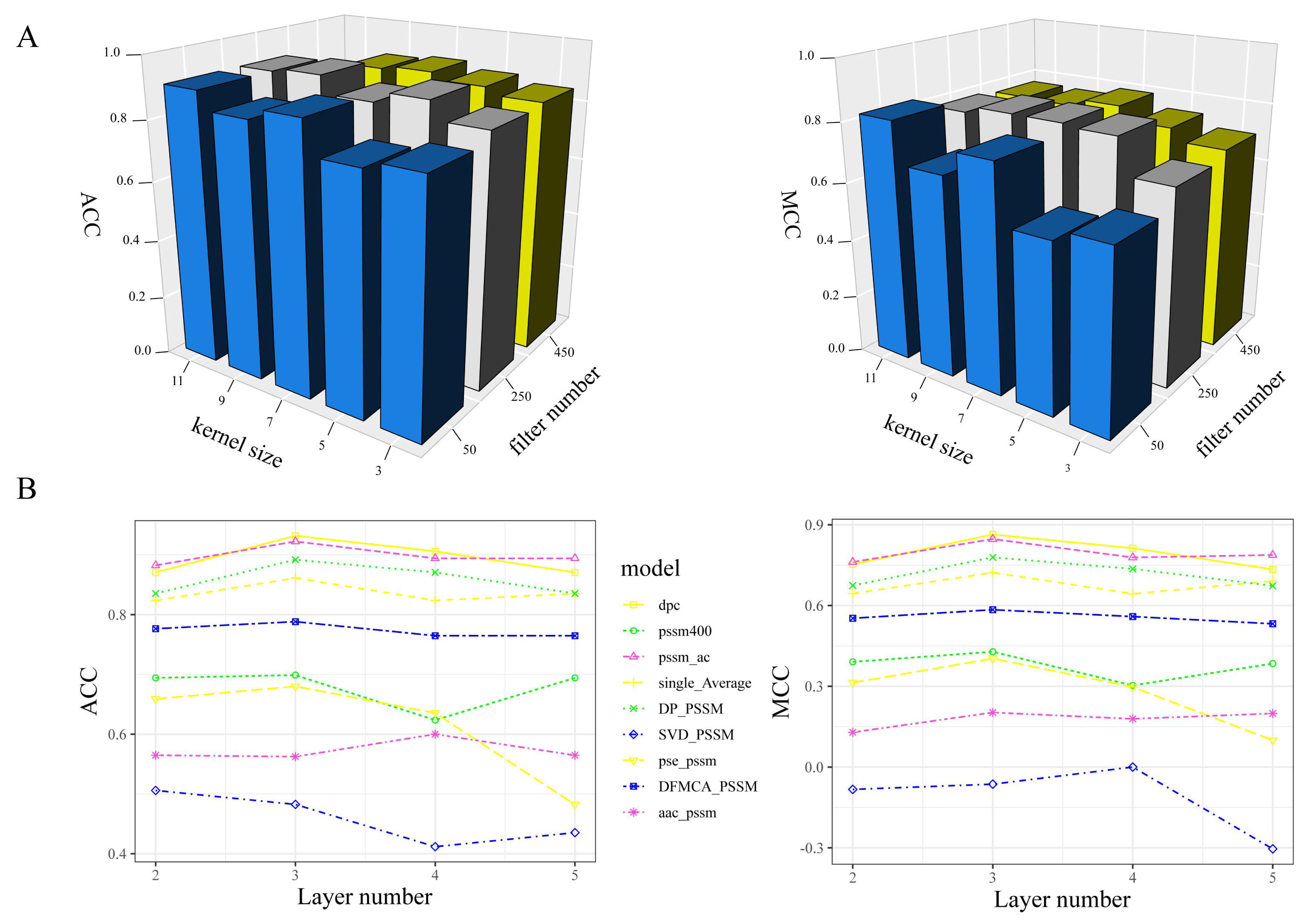

Supplement: S1 Fig — (A) Performance evaluation of CNN architectures across varying convolutional kernel sizes and filter counts. (B) Comparative analysis of DNN architectures using different protein descriptors and hidden layer configurations. (TIF) [file pone.0319329.s001.tif]
